# Growth Differentiation Factor 15 Is Associated with Platelet Reactivity in Patients with Acute Coronary Syndrome

**DOI:** 10.3390/jcm12041627

**Published:** 2023-02-17

**Authors:** David Mutschlechner, Maximilian Tscharre, Patricia P. Wadowski, Joseph Pultar, Constantin Weikert, Silvia Lee, Beate Eichelberger, Simon Panzer, Thomas Perkmann, Thomas Gremmel

**Affiliations:** 1 Department of Internal Medicine I, Cardiology and Intensive Care Medicine, Landesklinikum Mistelbach-Gänserndorf, 2130 Mistelbach, Austria; 2 Department of Internal Medicine, Cardiology and Nephrology, Landesklinikum Wiener Neustadt, 2700 Wiener Neustadt, Austria; 3 Department of Internal Medicine II, Medical University of Vienna, 1090 Vienna, Austria; 4 Department of Anesthesia and Intensive Care Medicine, Universitätsklinikum St. Pölten, 3100 St. Pölten, Austria; 5 Department of Blood Group Serology and Transfusion Medicine, Medical University of Vienna, 1090 Vienna, Austria; 6 Department of Laboratory Medicine, Medical University of Vienna, 1090 Vienna, Austria; 7 Institute of Cardiovascular Pharmacotherapy and Interventional Cardiology, Karl Landsteiner Society, 3100 St. Pölten, Austria

**Keywords:** growth differentiation factor 15, acute coronary syndrome, prasugrel, ticagrelor, platelet reactivity

## Abstract

Bleeding events in patients with acute coronary syndrome (ACS) are a risk factor for adverse outcomes, including mortality. We investigated the association of growth differentiation factor (GDF)-15, an established predictor of bleeding complications, with on-treatment platelet reactivity in ACS patients undergoing coronary stenting receiving prasugrel or ticagrelor. Platelet aggregation was measured by multiple electrode aggregometry (MEA) in response to adenosine diphosphate (ADP), arachidonic acid (AA), thrombin receptor-activating peptide (TRAP, a protease-activated receptor-1 (PAR-1) agonist), AYPGKF (a PAR-4 agonist) and collagen (COL). GDF-15 levels were measured using a commercially available assay. GDF-15 correlated inversely with MEA ADP (r = −0.202, *p* = 0.004), MEA AA (r = −0.139, *p* = 0.048) and MEA TRAP (r = −0.190, *p* = 0.007). After adjustment, GDF-15 was significantly associated with MEA TRAP (*β* = −0.150, *p* = 0.044), whereas no significant associations were detectable for the other agonists. Patients with low platelet reactivity in response to ADP had significantly higher GDF-15 levels (*p* = 0.005). In conclusion, GDF-15 is inversely associated with TRAP-inducible platelet aggregation in ACS patients treated with state-of-the-art antiplatelet therapy and significantly elevated in patients with low platelet reactivity in response to ADP.

## 1. Introduction

Despite tremendous progress in the diagnosis and treatment of acute coronary syndromes (ACS), coronary heart disease still is the major cause of death worldwide [1]. 

Antithrombotic therapy is an essential part of the therapeutic regimen in ACS patients, both in acute treatment and in secondary prevention [2,3]. In large randomized clinical trials, the novel P2Y12 receptor antagonists ticagrelor and prasugrel have shown greater efficacy than clopidogrel in reducing ischemic outcomes [4,5,6]. Therefore, current guidelines recommend ticagrelor and prasugrel over clopidogrel in ACS patients after percutaneous coronary intervention (PCI) [7,8]. Despite their benefits in terms of preventing ischemic outcomes, the novel P2Y12 receptor antagonists are associated with a higher risk of bleeding complications compared with clopidogrel [4,5]. 

Growth differentiation factor (GDF)-15 is part of the transforming growth factor-β protein family and is associated with inflammation, metabolic distress and malignant diseases [9,10,11,12,13,14]. In patients with atrial fibrillation (AF), GDF-15 has been identified as a risk factor for bleeding events [14]. Therefore, the ESC guidelines for the diagnosis and management of AF have included GDF-15 in the biomarker-based ABC-bleeding risk score (age, biomarker and clinical history) [15]. Furthermore, in ACS patients, elevated GDF-15 levels were associated with an increased risk of major bleeding complications, as well as with ischemic events including cardiovascular mortality, but the underlying mechanisms are not fully understood [10,16]. In experimental models, GDF-15 has been demonstrated to affect hemostasis by preventing platelet integrin action, as well as thrombus formation [17,18,19]. Moreover, we recently demonstrated that, in patients with left ventricular assist devices (LVAD) on potent antithrombotic therapy, GDF-15 correlated inversely with platelet reactivity via protease-activated receptor-1 (PAR-1) [20]. 

However, no data on GDF-15 and platelet reactivity in ACS patients have been reported so far. We therefore investigated the association of GDF-15 with on-treatment platelet reactivity in a cohort of ACS patients undergoing PCI.

## 2. Materials and Methods

### 2.1. Study Population

The study cohort has been described previously [21]. In total, 206 ACS patients on daily aspirin (100 mg/day), and either prasugrel (10 mg/d, *n* = 116) or ticagrelor (180 mg/d, *n* = 90), were included. Pre- and periprocedurally, all patients received weight-adjusted unfractionated heparin (UFH) (70–100 IU/kg, aiming for an activated clotting time > 250 s) [22]. None of the patients received a GPIIb/IIIa inhibitor. After successful PCI, blood was drawn after an overnight fast. Exclusion criteria included oral anticoagulation with either vitamin K antagonists (warfarin, phenprocoumon and acenocoumarol) or direct oral anticoagulants (edoxaban, dabigatran, apixaban and rivaroxaban), a known aspirin, prasugrel or ticagrelor intolerance (allergic reaction and gastrointestinal bleeding complication), a history of bleeding complications or a positive family history of bleeding complications, treatment with ticlopidine, dipyridamole or nonsteroidal antirheumatic drugs, malignant myeloproliferative disorders or heparin-induced thrombocytopenia, major surgery within one week before enrollment, severe hepatic failure with impaired hepatic synthesis (spontaneous international normalized ratio [INR] ≥1.5 and albumin levels <35 mg/dl) [23], known qualitative defects in platelet function, a platelet count < 100.000 or > 450.000/µL and a hematocrit < 30%.

The study protocol was in accordance with the criteria of the Declaration of Helsinki and has been approved by the Ethics Committee of the Medical University of Vienna (EC-No. 1940/2013). All study participants gave written informed consent. 

### 2.2. Blood Sampling

Blood was drawn by aseptic venipuncture from an antecubital vein using a 21-gauge butterfly needle (0.8 × 19 mm; Greiner Bio-One, Kremsmunster, Austria) as previously described [24]. To avoid procedural deviations, all blood samples were taken by the same physician applying a light tourniquet that was immediately released, and the samples were mixed by gently inverting the tubes.

### 2.3. Multiple Electrode Aggregometry (MEA)

As described previously, whole blood impedance aggregometry was performed with the Multiplate analyzer (Roche Diagnostics, Mannheim, Germany) [21,25,26]. 

One Multiplate test cell contains two independent sensor units. One unit consists of two silver-coated highly conductive copper wires with a length of 3.2 mm. After dilution (1:2 with 0.9% NaCl solution) of hirudin-anticoagulated whole blood and stirring in the test cuvettes for 3 min at 37 °C, adenosine diphosphate (ADP, 6.4 μM), acetylsalicylic acid (AA, 0.5 mM), thrombin receptor-activating peptide (TRAP, a protease-activated receptor [PAR]-1 agonist; 32 μM) and collagen (COL, 2.7 μg/mL) or AYPGKF (a PAR-4 agonist, 645 μM, all from Roche Diagnostics, Mannheim, Germany) were added. Aggregation was then continuously recorded for 6 min. The concentrations of all agonists were chosen according to the manufacturer’s recommendations. The adhesion of activated platelets to the electrodes led to an increase in impedance, which was detected for each sensor unit separately and transformed to aggregation units (AU) that were plotted against time. The AUs at 6 min were used for calculations. One AU corresponds to 10 AU*min (area under the curve of AU) [21].

### 2.4. Growth Differentiation Factor 15 (GDF)-15

As previously described, GDF-15 was measured on a Cobas^®^ e602 modular analyzer (Roche Diagnostics, Mannheim, Germany) according to the manufacturer’s instructions using the CE-marked Roche Elecsys^®^ GDF-15 electrochemiluminescence sandwich immunoassay (ECLIA) (Roche Diagnostics, Rotkreuz, Switzerland) [20].

### 2.5. Statistical Analysis

All continuous variables are expressed as median (interquartile range (IQR)). Categorical variables are given as numbers (%). Continuous variables were compared by Mann–Whitney U-test. χ2-tests were performed for comparison of categorical variables. Spearman rank correlation was used to test for correlations. Multivariable linear regression analyses using a stepwise inclusion algorithm were used to adjust for patient characteristics. Covariates for adjustment were selected based on univariate analyses (*p* value ≤ 0.1). A significance level of 2-tailed *p* ≤ 0.05 was considered statistically significant. All statistical analyses were performed with SPSS 28.0.1.1. (Armonk, NY, USA).

## 3. Results

In total, 206 patients were available for final analysis. Baseline characteristics of the study cohort are shown in Table 1. Median age was 58 (IQR 50–66) years and 165 patients (80.1%) were male. One hundred thirty-five patients (65.5%) had an ST-elevation myocardial infarction (STEMI) and 69 patients (33.5%) had a non-ST-elevation myocardial infarction (NSTEMI). Median GDF-15 levels were 1201.5 pg/mL (IQR 865.0–1775.0 pg/mL).

**Figure 1:**
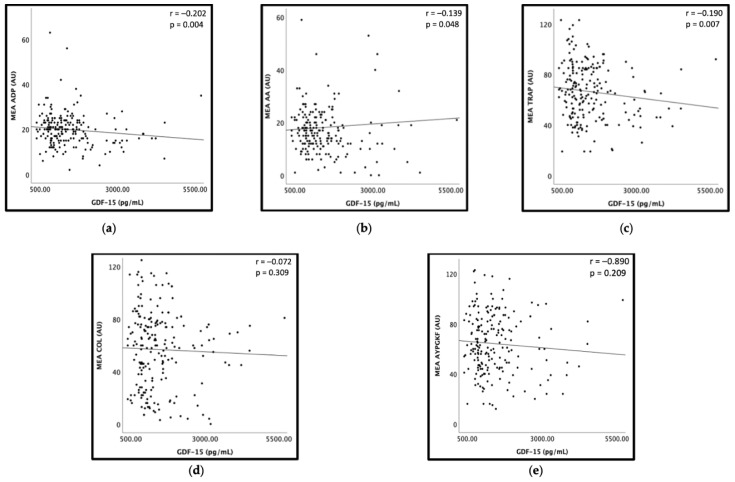
Correlations of GDF-15 with platelet aggregation by multiple electrode aggregometry (MEA). (**a**) Scatter plot showing GDF-15 (x-axis) versus adenosine diphosphate (ADP)-inducible platelet aggregation by MEA (y-axis). (**b**) Scatter plot showing GDF-15 (x-axis) versus arachidonic acid (AA)-inducible platelet aggregation by MEA (y-axis). (**c**) Scatter plot showing GDF-15 (x-axis) versus thrombin receptor-activating peptide (TRAP)-inducible platelet aggregation by MEA (y-axis). (**d**) Scatter plot showing GDF-15 (x-axis) versus collagen (COL)-inducible platelet aggregation by MEA (y-axis). (**e**) Scatter plot showing GDF-15 (x-axis) versus AYPGKF-inducible platelet aggregation by MEA (y-axis).

GDF-15 correlated inversely with MEA ADP (r = −0.202, *p* = 0.004), MEA AA (r = −0.139, *p* = 0.048) and with MEA TRAP (r = −0.190, *p* = 0.007), whereas no significant correlation of GDF-15 with MEA COL (r = −0.072, *p* = 0.309) and MEA AYPGKF (r = −0.890, *p* = 0.209) was detectable (Figure 1). In a linear regression model, MEA TRAP was independently associated with GDF-15 (β = −0.150; *p* = 0.044; Table 2).

MEA ADP and MEA AA did not remain significantly associated with GDF-15 (Table 3 and Table 4).

High on-treatment residual platelet reactivity (HRPR) in response to AA (HRPR AA) and ADP (HRPR ADP) and low platelet reactivity (LPR) were defined according to previous studies [24,27]. The respective cut-off values for HRPR were AU ≥20 for HRPR AA and AU ≥46 for HRPR ADP. The respective cut-off value for LPR ADP was ≤18 AU. With the use of these thresholds, HRPR AA was detected in 65 patients (31.6%), and HRPR ADP was detected in 3 patients (1.5%). LPR to ADP was present in 109 patients (52.9 %) [24,27].

**Figure 2:**
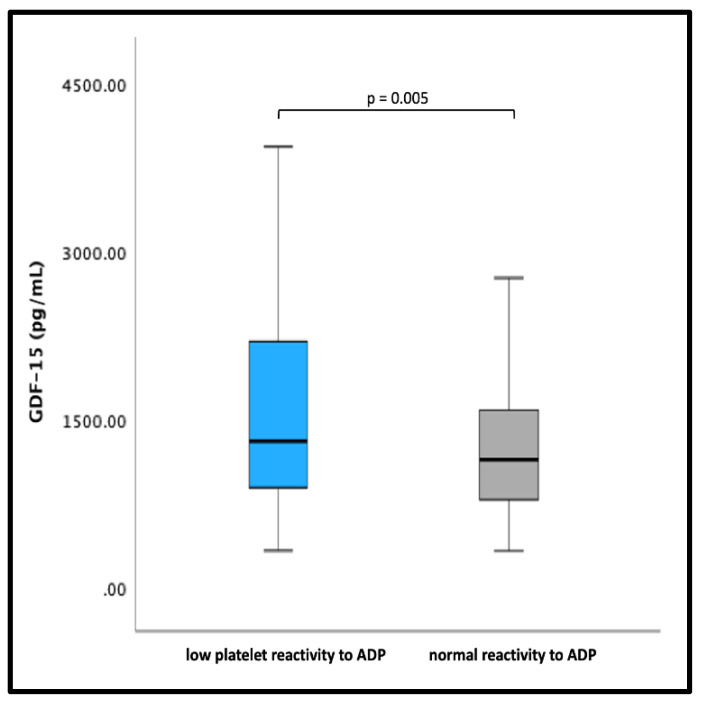
GDF-15 levels in patients with normal or high platelet reactivity versus low platelet reactivity to adenosine diphosphate (ADP). The boundaries of the box show the lower and upper quartiles, the line inside the box represents the median. Whiskers were drawn from the edge of the box to the highest and lowest values that are outside the box but within 1.5 times the box length.

There were no significant differences in GDF-15 levels between patients with HRPR AA and HRPR ADP compared with patients with normal platelet reactivity (all *p* > 0.05). Of note, patients with LPR ADP had significantly higher GDF-15 levels than patients without LPR ADP (1333 pg/mL [IQR 934–2468] vs. 1181 pg/mL [IQR 828–1580], *p* = 0.005) (Figure 2).

## 4. Discussion

The present study is the first to investigate the association of GDF-15 levels with on-treatment platelet reactivity in ACS patients undergoing PCI on potent antithrombotic therapy with prasugrel or ticagrelor. In our cohort, GDF-15 levels inversely correlated with ADP-, AA- and TRAP-inducible platelet aggregation measured by MEA. After multivariable adjustment, GDF-15 remained significantly associated with MEA TRAP. Moreover, patients with LPR in response to ADP had significantly higher GDF-15 levels.

MEA is a readily available and highly specific assay for measuring agonist-inducible platelet aggregation as an impedance increase between two electrodes [28]. In previous studies, HRPR ADP as assessed by MEA was associated with ischemic events after PCI, whereas LPR ADP was associated with an increased rate of bleeding complications [27,29,30,31].

GDF-15 has been linked to bleeding complications in several cohorts. In the ARISTOTLE (Apixaban for Reduction in Stroke and Other Thromboembolic Events in Atrial Fibrillation) trial, GDF-15 was shown to be an independent risk factor not only for major bleeding complications but also for mortality and stroke in AF patients randomized to either warfarin or apixaban [14]. Due to its association with major bleeding, GDF-15 found its way into the ABC-bleeding risk score for AF patients [15]. Moreover, previous studies have demonstrated a correlation of GDF-15 with bleeding complications in patients with coronary artery disease on dual antiplatelet therapy (DAPT) [14,16]. In the PLATO (PLATelet inhibition and patient Outcomes) trial, GDF-15 was an independent risk factor for major bleeding across different bleeding locations, as well as for the composite endpoint of cardiovascular death, spontaneous myocardial infarction and stroke in ACS patients. Moreover, it was shown that risk stratification for cardiovascular mortality and major bleeding could be improved by adding GDF-15 in addition to other established risk factors [16].

However, little is known about the underlying mechanisms of the association of GDF-15 with bleeding complications. Several explanations have been proposed so far: First, GDF-15 is associated with numerous cardiovascular risk factors, including age, comorbidities and various biomarkers, which might increase bleeding risk [10,16]. Second, GDF-15 has been proposed as a marker of cellular stress and vulnerability [14,16]. Finally, GDF-15 interferes with integrin activation in experimental models. Via the activation of Cdc42 and by inhibiting the GTPase Rap-1, GDF-15 reduces activation and clustering of β2-integrins [11,17]. In GDF-15 knock-out mice, platelet aggregation in response to ADP and U46619 (a thromboxane A2 receptor agonist) was increased compared with wild-type mice [17]. Moreover, GDF-15 impaired ADP-, thrombin- and U46619-inducible platelet β1- and β3-integrin activation. In contrast, GDF-15 did not affect GPIb or platelet collagen receptors. Our results confirm these experimental data, as we report an inverse association of GDF-15 with TRAP-inducible platelet aggregation in ACS patients undergoing PCI. Moreover, patients with LPR in response to ADP, a known risk factor for bleeding complications, had significantly higher GDF-15 levels. Also in line with former reports, no correlation of GDF-15 with COL- and AYPGKF-inducible platelet reactivity was detectable. Our data are further supported by a recent report of our working group demonstrating an inverse correlation of GDF-15 with activated GPIIb/IIIa expression via PAR-1 and with PAR-1-mediated platelet reactivity measured by MEA in patients with LVADs receiving aspirin and a vitamin-K antagonist. In this former study, there was also a linear but nonsignificant association of GDF-15 with platelet activation and aggregation in response to ADP [20].

Our results suggest that GDF-15 may help to identify ACS patients at an increased bleeding risk who may benefit from a less aggressive antithrombotic treatment regimen following PCI. This is particularly important, as ACS patients’ bleeding events are associated with an increased risk of major adverse cardiac events (MACE) and death [32,33,34,35].

Unfortunately, we did not include a control group of patients without ACS. Consequently, our results only apply to ACS patients. However, as previously demonstrated, there is also an inverse association of GDF-15 with TRAP-inducible platelet reactivity in LVAD patients [20]. These previous findings, together with the current observations, strengthen the hypothesis that high GDF-15 levels may be associated with decreased platelet aggregation. Nevertheless, additional studies in healthy individuals and patients with cardiovascular disease are needed to further investigate the association of GDF-15 with platelet reactivity and to reveal underlying mechanisms, as well as the clinical relevance of these findings. Furthermore, due to limited follow-up data, no outcome analyses on bleeding complications were performed in the present study cohort. Accordingly, further prospective studies are necessary to determine whether GDF-15 is a valuable biomarker for bleeding risk stratification in ACS patients, similar to the ABC bleeding risk score for AF patients [15],

Alternatively, due to its association with lower TRAP-inducible platelet aggregation, high GDF-15 itself may to some extent prevent MACE, since high PAR-1-mediated platelet activation is linked to an increased risk of ischemic outcomes [36].

## 5. Conclusions

GDF-15 is inversely associated with TRAP-inducible platelet aggregation in ACS patients treated with state-of-the-art antiplatelet therapy and significantly elevated in patients with LPR in response to ADP. GDF-15 may help to identify ACS patients at an increased risk of bleeding who may benefit from less aggressive antithrombotic therapy.

## Figures and Tables

**Table 1:** Baseline characteristics of prasugrel- and ticagrelor-treated patients.

Characteristics	Prasugrel (*n* = 116)	Ticagrelor (*n* = 90)	*p*
GDF-15, pg/mL	1136 [865–1564]	1492 [868–2027]	0.014
Age, years	56 [48–64]	59 [51–70]	0.011
Male patients, No. (%)	94 (81.0)	71 (78.9)	0.702
Body mass index, kg/m^2^	27.7 [25.2–31.0]	26.8 [24.3–29.7]	0.293
Prior myocardial infarction, No. (%)	18 (15.5)	15 (16.7)	0.762
Prior stroke or TIA, No. (%)	4 (3.4)	2 (2.2)	0.647
Arterial hypertension, No. (%)	74 (63.8)	61 (67.8)	0.510
Hyperlipidemia, No. (%)	87 (75.0)	64 (71.1)	0.757
Peripheral artery disease, No. (%):	8 (6.9)	5 (5.6)	0.349
ACS, No. %			
STEMI	108 (93.1)	27 (30.0)	<0.01
NSTEMI	7 (6.0)	62 (68.9)	
Diabetes mellitus type II, No. (%):	24 (20.7)	27 (30.0)	0.097
Smoker, No. (%):	69 (59.5)	47 (52.2)	0.517
Serum creatinine, mg/dl	0.89 [0.76–1.01]	1.00 [0.82–1.17]	0.001
Platelet count, G/l	221 [194–251]	227 [191–269]	0.567
High-sensitivity CRP, mg/dL	1.35 [0.69–3.85]	1.29 [0.82–3.84]	0.299
Hemoglobin, mmol/L	14.0 [13.2–14.9]	13.6 [12.7–14.6]	0.197
proBNP, pg/mL	748 [289–1494]	603 [220–1113]	0.096
Statin, No. (%)	114 (98.2)	88 (97.8)	0.855
Beta blocker, No. (%)	111 (95.7)	87 (96.7)	0.606
ACE inhibitor, No. (%)	97 (83.6)	70 (77.8)	0.569
ARB, No. (%)	16 (13.8)	17 (18.9)	0.318
Calcium channel blocker, No. (%)	10 (8.6)	8 (8.9)	0.942

Continuous data are shown as median (interquartile range). Dichotomous data are shown as *n* (%). ACE = angiotensin-converting enzyme; ACS = acute coronary syndrome; ARB = angiotensin-receptor blocker; CRP = C-reactive protein; NSTEMI = non-ST-elevation myocardial infarction; proBNP = probrain natriuretic peptide; STEMI = ST-elevation myocardial infarction.

**Table 2:** Unadjusted and adjusted linear regression for MEA ADP.

	Unadjusted		Adjusted	
	*β*	*p*	*β*	*p*
GDF-15	−0.171	0.013	−0.124	0.324
Age	−0.090	0.191		
Sex	0.088	0.202		
BMI	0.013	0.853		
Type of ACS	−0.013	0.853		
HLP	−0.061	0.376		
History of smoking	−0.091	0.192		
Arterial hypertension	0.047	0.499		
Previous MCI	0.095	0.174		
P2Y12 antagonist	−0.034	0.625		
CKD	−0.128	0.074	−0.024	0.843
Diabetes mellitus	0.121	0.085	0.065	0.407

Abbreviations: ACS = acute coronary syndrome; BMI = body mass index; CKD = chronic kidney failure; GDF-15 = growth differentiation factor-15; HLP = hyperlipidemia; MCI = myocardial infarction.

**Table 3:** Unadjusted and adjusted linear regression for MEA AA.

	Unadjusted		Adjusted	
	*β*	*p*	*β*	*p*
GDF-15	0.070	0.312		
Age	−0.083	0.228		
Sex	0.130	0.134		
BMI	−0.016	0.821		
Type of ACS	−0.128	0.065	−0.101	0.152
HLP	−0.045	0.520		
History of smoking	−0.149	0.032	−0.115	0.106
Arterial hypertension	0.123	0.077	0.088	0.217
Previous MCI	0.100	0.153		
P2Y12 antagonist	0.009	0.904		
CKD	0.094	0.192		
Diabetes mellitus	0.001	0.990		

Abbreviations: ACS = acute coronary syndrome; BMI = body mass index; CKD = chronic kidney failure; GDF-15 = growth differentiation factor-15; HLP = hyperlipidemia; MCI = myocardial infarction.

**Table 4:** Unadjusted and adjusted linear regression for MEA TRAP.

	Unadjusted		Adjusted	
	*β*	*p*	*β*	*p*
GDF-15	−0.200	0.004	−0.150	0.044
Age	−0.180	0.009	−0.029	0.712
Sex	−0.042	0.544		
BMI	0.038	0.589		
Type of ACS	−0.051	0.463		
HLP	−0.144	0.037	−0.149	0.031
History of smoking	−0.225	0.001	−2.116	0.036
Arterial hypertension	0.125	0.072	0.092	0.194
Previous MCI	0.084	0.231		
P2Y12 antagonist	0.042	0.552		
CKD	−0.096	0.181		
Diabetes mellitus	0.050	0.477		

Abbreviations: ACS = acute coronary syndrome; BMI = body mass index; CKD = chronic kidney failure; GDF-15 = growth differentiation factor-15; HLP = hyperlipidemia; MCI = myocardial infarction.

## Data Availability

The data underlying this article will be shared on reasonable request to the corresponding author.
